# HVint: A Strategy for Identifying Novel Protein-Protein Interactions in Herpes Simplex Virus Type 1*[Fn FN2]

**DOI:** 10.1074/mcp.M116.058552

**Published:** 2016-07-06

**Authors:** Paul Ashford, Anna Hernandez, Todd Michael Greco, Anna Buch, Beate Sodeik, Ileana Mihaela Cristea, Kay Grünewald, Adrian Shepherd, Maya Topf

**Affiliations:** From the: ‡Institute of Structural and Molecular Biology, Birkbeck College, University of London, Malet Street, London, WC1E 7HX, UK;; §Oxford Particle Imaging Centre, Division of Structural Biology, Wellcome Trust Centre for Human Genetics, University of Oxford, Oxford, OX3 7BN, UK;; ¶Department of Molecular Biology, Princeton University, Lewis Thomas Laboratory, Washington Road, Princeton, New Jersey 08544;; ‖Institute of Virology, Hannover Medical School, OE 4310, Carl-Neuberg-Str. 1, D-30623, Hannover, Germany

## Abstract

Human herpesviruses are widespread human pathogens with a remarkable impact on worldwide public health. Despite intense decades of research, the molecular details in many aspects of their function remain to be fully characterized. To unravel the details of how these viruses operate, a thorough understanding of the relationships between the involved components is key. Here, we present HVint, a novel protein-protein intraviral interaction resource for herpes simplex virus type 1 (HSV-1) integrating data from five external sources. To assess each interaction, we used a scoring scheme that takes into consideration aspects such as the type of detection method and the number of lines of evidence. The coverage of the initial interactome was further increased using evolutionary information, by importing interactions reported for other human herpesviruses. These latter interactions constitute, therefore, computational predictions for potential novel interactions in HSV-1. An independent experimental analysis was performed to confirm a subset of our predicted interactions. This subset covers proteins that contribute to nuclear egress and primary envelopment events, including VP26, pUL31, pUL40, and the recently characterized pUL32 and pUL21. Our findings support a coordinated crosstalk between VP26 and proteins such as pUL31, pUS9, and the CSVC complex, contributing to the development of a model describing the nuclear egress and primary envelopment pathways of newly synthesized HSV-1 capsids. The results are also consistent with recent findings on the involvement of pUL32 in capsid maturation and early tegumentation events. Further, they open the door to new hypotheses on virus-specific regulators of pUS9-dependent transport. To make this repository of interactions readily accessible for the scientific community, we also developed a user-friendly and interactive web interface. Our approach demonstrates the power of computational predictions to assist in the design of targeted experiments for the discovery of novel protein-protein interactions.

One important milestone toward understanding the complexity of viral infections is to unravel the interplay between viral proteins (the *intraviral interactome*). This is particularly important for complex and large DNA viruses, such as human herpesviruses, which have the ability to express a large number of viral gene-products. For example, the genome of HSV-1 encodes for more than 75 viral proteins, and the levels of these proteins are temporally and spatially regulated during the progression of the viral infection (1). Human herpesviruses cause life-long infections and many human and animal diseases. The severity of symptoms ranges from cold sores, genital ulcers, and blisters to blindness and life-threatening conditions, including fatal encephalitis, meningitis and cancer (2, 3). Infections from herpesviruses are also a major threat to immunosuppressed patients (*e.g.* infected by human immunodeficiency virus) and have been associated with Alzheimer's disease (3, 4).

Protein interactome studies can reveal critical biological information and shed light on mechanisms underlying infectious diseases (5), supporting proteome-wide annotation (6, 7) and the development of therapeutic strategies (8, 9). The current methods used for building protein-protein interaction (PPI) networks mainly rely on known interactions and sequence analysis (11–13). Recently the field has moved forward through the development of structural and functional proteomics techniques that include fluorescence microscopy and Mass Spectrometry (MS)-based approaches (6, 14–16). These techniques have helped to increase the coverage of the interactome in the context of infection. Several public repositories of PPI data exist, such as IntAct (www.ebi.ac.uk/intact/) (15). Multiple evidence lines, depending on the nature of the interaction itself and, importantly, the detection method used, can support each individual PPI. For example, evidence for PPIs can be derived from biochemical assays, such as Yeast Two-Hybrid (Y2H), CoImmunoprecipitation (Co-IP) ^1^, *in vitro* binding assays, and protein cross-linking, which can be then analyzed by MS. PPIs can also be derived from Nuclear Magnetic Resonance (NMR), x-ray crystallography and Electron Microscopy techniques. Most resources include evidence manually extracted from the literature. Furthermore, databases that are not explicitly dedicated to storing PPI data provide additional valuable resources, such as the Protein Data Bank (PDB www.rcsb.org/) (16) and the Electron Microscopy Data Bank (EMDB www.emdatabank.org/) (17), which contain structural information. Other databases gather PPIs based on information from multiple resources (*e.g.* VirHostNet www.virhostnet.prabi.fr/, STRING-DB http://string-db.org/) (20, 21). However, constructing a PPI network from disparate sources while ensuring trustworthiness and high coverage is challenging. Experiments vary in terms of both reliability and ability to discriminate between different categories of interactions, notably direct (physical) *versus* indirect interactions (*e.g.* proteins belonging to the same protein complex but without direct physical contact) as well as transient *versus* stable interactions (22). Moreover, for many nonmodel organisms, the number of known PPIs remains limited, and thus there is a need to develop hypotheses about additional PPIs that are not yet supported by direct experimental evidence.

Computational prediction of PPIs (19–23) provide the opportunity to maximize the coverage of interaction networks. These predictions often rely on sequence homology or machine learning methods (24–26). Several studies have now illustrated that transferring interaction data between close homologous species (*i.e. interologues mapping* (31)) is a suitable approach to expand PPI data for a given species. Computational methods for building and analyzing PPI networks have the potential to identify novel candidates for future experimental validation, thereby saving valuable time and resources (30, 32, 33).

In this study, we created HVint, a new database for intraviral PPIs for an important human pathogen, of herpes simplex virus type 1 (HSV-1, also known as HHV-1), and derived the associated PPI network. To accomplish this, PPIs reported for any stage of the “life cycle” were compiled, *i.e.* interaction data both for proteins that are incorporated within extracellular virion particles and proteins that are only expressed in infected cells has been integrated. Information on the location of these proteins within the different structural layers of the virion particle (34, 35), namely the capsid, the tegument and the viral envelope, was included in the annotation of the interactions in HVint. To further expand the HVint database, we integrated the subsets of data from existing databases for any stage of the virus “life cycle”, implemented a method for scoring multiple lines of experimental evidence, and incorporated homologous interactions from the other human herpesviruses of the α-, β-, and γ-herpesvirus subfamilies. As a result, our network has significantly higher coverage than previous HSV-1 networks derived from existing databases (17, 18, 20, 36, 37), and it predicts novel interactions. We validated several of these predictions by affinity purification-MS using primary human fibroblasts infected with an HSV-1 virus strain expressing the small capsid protein VP26 tagged with green fluorescent protein (EGFP). Lastly, we developed a user-friendly interactive web interface, which will allow the scientific community to readily access and analyze the interaction data. Taken together, this work demonstrates the value of data integration and homology transfer in predicting previously uncovered interactions in a complex virus such as HSV-1 and in guiding future experimental work.

## EXPERIMENTAL PROCEDURES

### 

#### 

##### HVint Data Integration

**Fig. 1.**
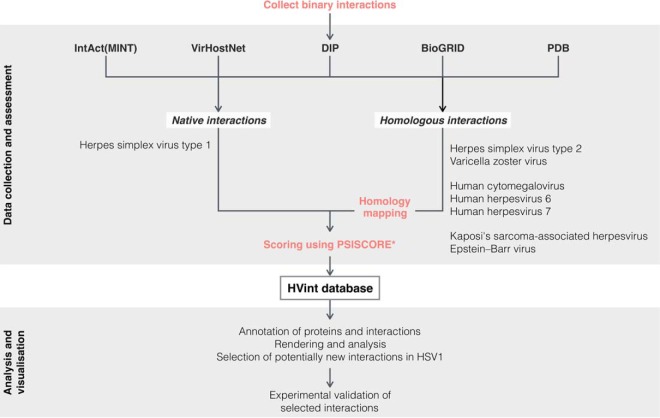
**Workflow of the procedure to generate and process protein-protein interaction data for the HVint database.**

**Fig. 2.**
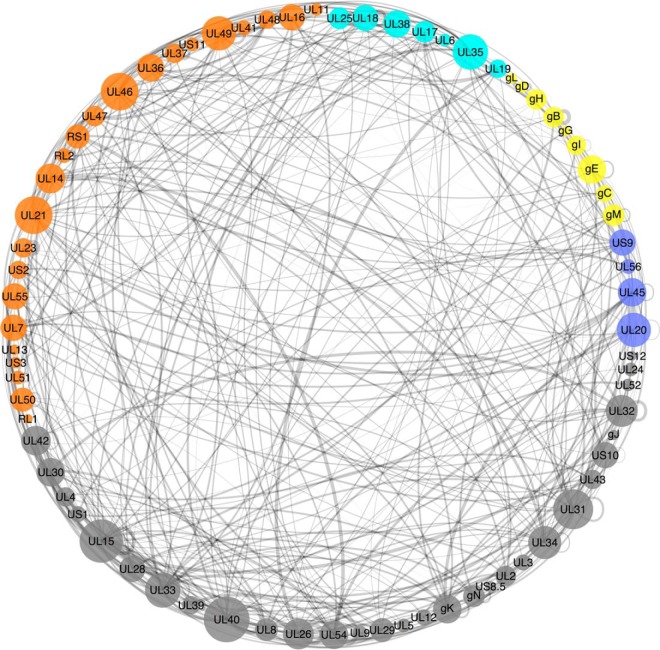
**Circular layout of the novel PPI network.** The respective node size indicates the number of interacting partners for each protein (*degree*). The edge width is scaled according to the confidence score associated with the interaction. Nodes are color-coded according to the respective protein location in the virion particles - gray: protein has not been detected in virions by MS in Loret *et al.* 2008 (34); cyan - capsid or capsid-associated protein; orange - tegument protein; yellow - envelope glycoprotein; dark blue - other envelope protein (not glycoproteins).

The PPI data compiled to generate our novel intra-viral protein interactome includes interactions identified by a range of experimental methods drawn from multiple sources (Figs. 1 and 2). First, data from four publicly available protein interaction databases, IntAct (15) (June 2015), VirHostNet 2.0 (18) (June 2015), Database of Interacting Proteins (DIP) (36) (October 2015) and BioGRID (37) (October 2015) were collected (38–47). Custom Perl scripts were used to parse the data and to select “native” interactions directly reported for HSV-1, and “homologous” interactions originally reported for any of the other human herpesviruses, *i.e.* herpes simplex virus type 2 (HSV-2, HHV-2), varicella-zoster virus (VZV, HHV-3), Epstein-Barr virus (EBV, HHV-4), human cytomegalovirus (HCMV, HHV-5), human herpesvirus 6 (HHV-6, A and B), human herpesvirus 7 (HHV-7), or Kaposi's sarcoma-associated herpesvirus (KSHV, HHV-8). The search was conducted using taxonomy identifiers (IDs) and all reported strains for each species were considered. Where possible, protein IDs, as reported in the above interaction databases, were mapped to UniProt (48) accession numbers using the UniProt ID *mapping* tool (*e.g.* DIP and BioGRID interactor IDs are from a variety of databases, including UniProt (48), NCBI RefSeq, and EMBL/GenBank/DDBJ). In addition we collected interactions derived from structural evidence provided by the Protein Data Bank (PDB) (16) (June 2015). The taxonomy browser in PDB was used to retrieve entries associated with human herpesvirus species. The resultant list was manually curated to discard entries containing a single protein, and considered both homo- and hetero-interactions between the components. Interactions obtained from this latter data set were also classified as native (from HSV-1) or homologous (from other human herpesvirus species) interactions according to their source species.

##### Homology Transfer of Interactions

Interactions gathered from other human herpesvirus species were included in HVint only if for a given interaction both interacting partners in the original species could be mapped to HSV-1 strain 17^+^ using the homology detection method described below. This same rationale was also applied to transfer interactions reported for any other strains of HSV-1 different from strain 17^+^. Homology relationships were defined based on the results of HHblits (49) (version 2.0.16). For each of the UniProt protein IDs involved in homologous species, its FASTA sequence was retrieved and used to generate a multiple sequence alignment against the entire UniProt Hidden Markov model (HMM) database (uniprot20_2012_10) with the number of iterations set to 3. An in-house Perl script was used to extract, where possible, the highest confidence match between the query and a protein in HSV-1 (any strain). The resulting HSV-1 UniProt IDs were mapped to HSV-1 strain 17^+^ (UniProt reference proteome for HSV1) via UniRef90 clusters (50) with in-house scripts.

The distribution of the experimental detection methods defining the interactions in our database is summarized in supplemental Fig. S1. Y2H methods represent the largest fraction followed by coimmunoprecipitation techniques, with more than 90% of the interactions detected so far solely by Y2H experiments. However, about 50% of the interactions (213 out of 419) are supported by two or more lines of evidence.

Based on prior knowledge, interactions in the network were also classified as interactions in the extracellular virions, *i.e.* taking place in extracellular viral particles, *versus* other interactions occurring during the viral lifecycle based on the classification of each of the interacting partners (34) (supplemental Fig. S2). All network figures were rendered using Gephi (51).

##### Scoring of Interaction Data

To measure the confidence of each of the interactions in HVint, we adapted a heuristic scoring method, namely MIscore (52, 53). MIscore was chosen because its effectiveness as a scoring function capable of combining diverse factors that affect the reliability of molecular interaction data is well established (53–55). First, scores are assigned to each individual piece of evidence for a given interaction. Aspects such as the interaction detection method(s), the type of interaction (*e.g.* colocalization or physical association) and the number of separate studies reporting the interaction, are taken into account. To this end, each evidence line is rigorously annotated with the PSI-MI ontology (56), a controlled vocabulary developed by the Proteomics Standards Initiative (PSI) (57) for the standardization of molecular interaction (MI) annotation data (example tags are, MI:009 for “protein complementation assay” and MI:0428 for “imaging technique”). Next, scores from different lines of evidence are brought together to compute a single value per interaction. A more detailed description of MIscore can be found in (53). In our study, we modified the original MIscore function. The evidence inferred from homology was scaled according to the sequence identity between HSV-1 and the corresponding homologs, as follows: given a pair of interacting sequences *A* and *B* and their HSV-1 homologs *A′* and *B′*, the original (unmodified) MIscore was multiplied by a scaling factor *s* defined as:

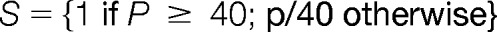
 where *p* is min{sequence identity between *A* and *A′*, sequence identity between *B* and *B′*}. In other words, the score was scaled according to the lowest percentage identity between either of the interacting partners with their respective homologs. Here 40% was chosen as a conservative cut-off, taking into account prior studies of homology and the conservation PPIs between related species (53). The resulting modified MIscore scores range between 0 and 1 (supplemental Fig. S3).

##### Network Analysis

We next assessed how filtering out interactions based on MIscore cut-offs affected the network topology by measuring how several network properties varied as interactions with a score below a certain cut-off were removed from the network. Self-interactions were not considered during this analysis, as they do not affect most of the network properties under study. We also analyzed how the network changes when separating direct *versus* indirect interactions according to the PSI-MI classification.

##### Experimental Validation by Affinity Purification-Mass Spectrometry

We used the HSV1(17^+^)blueLox-GFPVP26 strain, here denoted HSV1-GFPVP26, in which the small capsid protein VP26 has been tagged at the N terminus with EGFP (58). As a control, we used the HSV1(17^+^)blueLox-pMCMVGFP strain, here denoted HSV1-GFP, which expresses EGFP alone, under the control of the murine cytomegalovirus promoter, inserted between the UL55 and UL56 ORFs (59). The viruses were propagated, isolated, and titered in Vero cells (ATCC CCL81) grown in DMEM containing 10% FBS and 1% penicillin/streptomycin, as previously described (60, 61). Primary human foreskin fibroblast cells (HFFs) were infected with HSV-1 strains at 1 plaque forming unit per cell (pfu/cell) for fluorescence microscopy or 5 pfu/cell for immunoaffinity purification-MS studies. The immunoaffinity purifications of GFPVP26 or EGFP during productive HSV-1 infection were accomplished using magnetic beads conjugated with in-house generated rabbit anti-GFP antibodies, as previously described (60, 62). However, for the present work, the HSV-1 strain background was 17^+^, and not KOS as in our previous work. Furthermore, to capture additional interactions, we selected a cell lysis/immunoisolation buffer of intermediate stringency, namely 20 mm HEPES-KOH, pH 7.4, containing 0.11 m potassium acetate, 2 mm MgCl_2_, 0.1% Tween 20, 1 μm ZnCl_2_, 1 μm CaCl_2_, 0.75% Triton X-100, 200 mm NaCl, 0.2% deoxycholate, and 1/100 v/v protease inhibitor mixture (Sigma). The immunoaffinity purifications of GFPVP26 and GFP were each performed in three biological replicates.

After immunoisolation of GFPVP26 and its interacting partners, the proteins were separated (∼ 3 cm) by SDS-PAGE using a 4–12% Bis-Tris NuPAGE gel (∼3 cm), then stained with Coomassie blue and cut into gel slices. Gel slices were pooled to maintain approximate equal protein amounts (based on Coomassie blue staining) and digested in-gel with trypsin, as described (60). Extracted peptides from neighboring gel fractions were pooled (*n* = −6 fractions), desalted using SDB-RPS StageTips (63), concentrated to near dryness, then resuspended in 10 μl of 0.1% formic acid and analyzed by nanoliquid chromatography-tandem MS (nLC-MS/MS).

Briefly, peptides (4 μl) were separated by reverse phase chromatography-nLC (Acclaim PepMap RSLC, 1.8 μm × 75 μm x 50 cm) at a flow rate of 250 nL/min over a 90 min ACN gradient from 4–40% mobile phase B (A, 0.1% FA; B, 97% ACN in 0.1% FA) and ionized by ESI directly into the mass spectrometer (LTQ Orbitrap Velos equipped with an EASY-Spray ion source). The mass spectrometer was operated in a data-dependent acquisition mode. Each acquisition cycle comprised a single full-scan mass spectrum (*m*/*z* = 350–1700) in the orbitrap (*r* = 30,000 at *m*/*z* = 400) followed by CID MS/MS of the top 15 most abundant ions, with dynamic exclusion enabled.

MS/MS fragmentation spectra were extracted and peptide spectrum matches were obtained using Proteome Discoverer (ver. 1.4)/SEQUEST (ver 1.3) by searching independently against the forward and reverse subset of the human UniProt-SwissProt database (2013–08) appended with herpesvirus sequences and common contaminants (22,910 sequences). SEQUEST search parameters were defined as the following: full trypsin specificity, maximum of 2 missed trypsin cleavages, ion precursor mass tolerance of 10 ppm, fragment ion mass tolerance of 0.5 Da, fixed modification of cysteine carbamidomethylation, variable modifications of methionine oxidation, and serine, threonine, and tyrosine phosphorylation. The Proteome Discoverer workflow also included the precursor ions area detector node for MS1-based label-free quantification. Database search results were analyzed in Scaffold (ver. 4.6, Proteome Software, Inc) using the LFDR algorithm and a refinement search using the X! Tandem algorithm (Beavis Informatics), which specified the following additional modifications using the same search parameters as above: deamidation of glutamine and asparagine, acetylation of protein N termini. The global peptide and protein FDR were controlled to ≤ 1%, estimated by reverse database search matches. The MS proteomics data have been deposited to the ProteomeXchange Consortium via the PRIDE (64) partner repository with the data set PXD003599 and 10.6019/PXD003599 identifier. Unweighted protein spectrum counts and corresponding precursor area quantitative values were exported to Excel and additional filtering of the protein identifications was performed. Briefly, protein groups with ≥ 10 spectra in at least two out of three replicates were retained. For proteins identified in both GFPVP26 and the GFP controls, the spectral count fold enrichment was calculated for each biological replicate as the ratio of the spectral counts in each GFPVP26 replicate and the averaged GFP samples. Viral proteins with an average enrichment ratio of ≥ fivefold were retained. The Top3 precursor area method, which uses the three most intense peptides as a measure of protein concentration (65), was used to estimate the stoichiometry of viral proteins coisolated with GFPVP26.

##### Fluorescence Microscopy

Primary human fibroblast cells were cultured as described above, except on glass cover slips, and infected with HSV1-GFPVP26 at a multiplicity of infection of 1 pfu/cell. At 14 h postinfection, cells were fixed with 2% (v/v) paraformaldehyde in PBS for 15 min at room temperature and prepared for imaging as previously described (60). Nuclei were stained with DAPI. The subcellular localization of GFPVP26 was analyzed using a confocal microscope (Leica SP5) equipped with a 100x oil-immersion lens.

##### Experimental Design and Statistical Rationale

The HVint database was populated with non-redundant PPI data collated from five different public resources (IntAct, VirHostNet 2.0, DIP, BioGRID and PDB). In doing so, we distinguished between two different sets of PPI data: (1) PPIs directly reported in HSV-1 and (2) PPIs reported in other human herpesviruses species. HHblits was used to establish orthology-relationships between proteins interacting in homologous human herpesviruses and HSV-1 proteins. When HSV-1 homologous proteins were detected for both interacting partners in the source species, the interaction was transferred to the HSV-1 interactome and included in the HVint database. All PPI data in HVint was scored under the MIscore scheme, a heuristic scoring method designed to capture the heterogeneity of PPI data. MIscore is computed taking into account the annotation data for a given interaction. This annotation is defined by the HUPO PSI controlled vocabulary, which has been implemented by the public database members of the IMEx consortium (56). The final normalized scores result from the weighted sum of three different terms representing (1) the number of different citations reporting the interaction, (2) the corresponding detection method(s) used, and (3) the nature of the interaction (type). An earlier study compared the performance of MIscore with that of the normalized score implemented in Mentha, a large integrative database (not containing viral interactomes) with over 570,000 PPIs (66). It was tested on a benchmark of 500 high confidence human PPIs (positive data set) and 397 noninteractions extracted from Negatome (negative data set) (67). Around the optimal score cut-offs for each database (0.485 for MIscore, 0.343 for the Mentha score) MIscore was shown to have greater accuracy, precision, and recall than the Mentha score (0.76 *versus* 0.67, 0.70 *versus* 0.66 and 0.98 *versus* 0.85, respectively) (53). Our predicted interactions transferred from other human herpesviruses were scaled down from their originally assigned MIscore according to the sequence identity of each interacting protein to its corresponding HSV-1 homolog. The resulting scores take values between 0 and 1 (inclusive), with larger values reflecting higher confidence in the interaction. This calibrated scale was designed to allow users to fine-tune the selection of PPI data subsets in the database, based on their research purposes.

A subset of computationally predicted interactions was selected as potential candidates for experimental validation by IP-MS. To identify this subset, we studied how several parameters characterizing the topology of the interactome changed when interactions scoring below a given MIscore threshold were removed from the network. The changes in network parameters were assessed in the entire range between 0 to 1 MIscore thresholds, in steps of 0.1. The IP-MS experiments isolating HSV1-GFPVP26 were performed in three biological replicates and compared against three biological IP-MS replicates of HSV1-GFP. In total six biological samples were subjected to GeLC-MS/MS analysis.

## RESULTS

### 

#### 

##### HVint Interactome

**Fig. 3.**
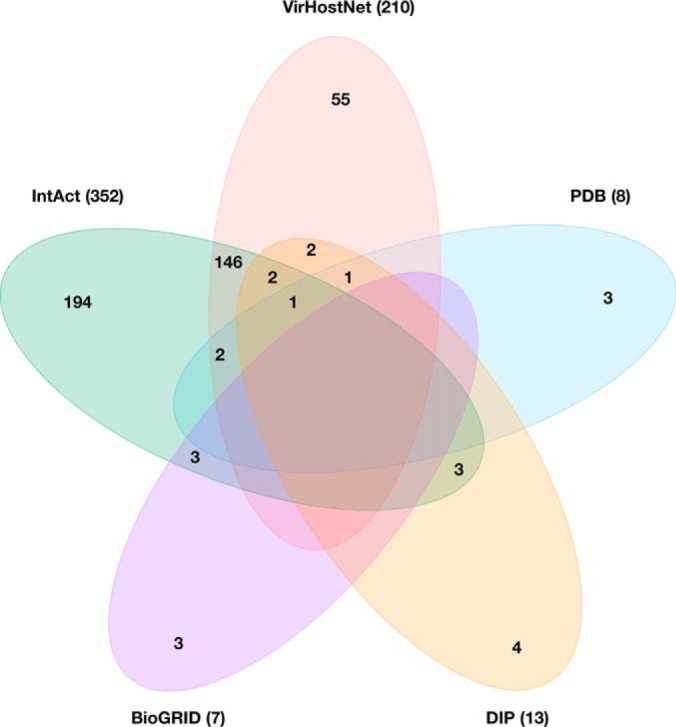
**Venn diagram showing the total number of non-redundant PPIs in the source public databases, *i. e.* IntAct (6–2015), VirHostNet (VHN; 6–2015), Database of Interacting Proteins (DIP; 10–2015), BioGRID (10–2015), and PDB (6–2015).** Numbers in parentheses refer to the total number of interactions originating from each source database. Numbers inside the colored areas indicate the degree of overlap between data sets. Empty subsets indicate zero overlap.

**Fig. 4.**
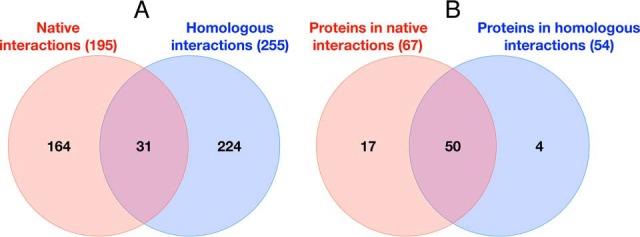
***A*, Venn diagram showing the total number of non-redundant PPIs in the novel HVint database** classified as native and homologous interactions, respectively. The numbers in parenthesis refer to the total number of PPIs; the numbers inside the circles indicate the degree of data set overlap. *B*, Venn diagram showing the number of non-redundant proteins involved in PPIs in the HVint database. Proteins are involved in native and/or homologous interactions. The numbers in parenthesis refer to the number of non-redundant proteins in the respective data set with the numbers inside the circles indicating the degree of data set overlap.

The computational and experimental pipeline developed in this work (see Experimental Procedures, Fig. 1) resulted in a novel HVint interactome (Fig. 2). The data fed into the pipeline originated from five different resources (IntAct, VirHostNet, DIP, BioGRID, and PDB) (Figs. 1 and 3). PPIs reported for HSV-1 and all of the other human herpesvirus species currently known (herpes simplex virus type 2, varicella-zoster virus, Epstein-Barr virus, human cytomegalovirus, human herpesviruses 6 and 7, and Kaposi's sarcoma-associated herpesvirus) were collected. The latter interactions were transferred to the HSV-1 interactome only when orthologs for both interacting partners in the source species (*e.g.* HCMV) could be found in the HSV-1 proteome. The resulting network comprises 73 nodes and 419 PPIs (including 36 self-interactions), the reliability of which was assessed with a modified MIscore function (see Methods). The confidence values for the interactions in the network range from 0.147 to 0.972. Confidence values were computed using a highly integrative and experimentally based scoring scheme that takes into account the type of interaction and detection method used to detect the interaction, as well as the number of different studies providing support for the interaction. Interactions were divided into two different data sets according to the source species: native interactions (reported in HSV-1) and homologous interactions (transferred from another human herpesvirus species). Homologous interactions (255 in total) account for ∼55% of the network, hence significantly increasing the size of the network beyond that of the native network (Fig. 4*A*). Twenty-nine interactions are present in both native and homologous PPI data sets, indicating conservation of PPIs between two or more herpesvirus species. These conserved herpesvirus PPIs involve 50 of the HSV-1 proteins, which correspond to about 70% of the HSV-1 proteome (Fig. 4*B*). 98% (413) of these interactions were classified as “direct interaction” or “physical association” by the PSI-MI annotation, meaning the respective experimental conditions indicate that this interaction either involves direct physical contact or that or the molecules are in close proximity.

##### New Interactions

Predicted PPIs that so far have not been supported by experimental evidence are of particular interest, as they point to putative biological mechanisms yet to be confirmed. To investigate whether such predicted PPIs indeed exist for HSV-1, a subset of high confidence *homologous* interactions was subjected to experimental validation. This subset was selected from the interactions in HVint having a confidence score greater than 0.4. The rationale behind this cutoff was that the initially observed topological properties of the interactome were barely altered for confidence score cut-offs in the range between 0 and 0.4 (supplemental Fig. S4), but for cut-offs between 0.4 and 0.5 drastic changes in several network parameters (including clustering coefficient, centralization, density, and average node degree) were observed. This calculated MIscore threshold is consistent with previously reported estimates of optimal cut-offs for scores predictions, within the range of 0.4 to 0.5 (53). We then retained PPIs derived from homology predictions only (*i.e.* only in the homologous data set). By definition, these homology-based PPIs have been reported for other human herpesviruses but not yet for HSV-1.

**Fig. 5.**
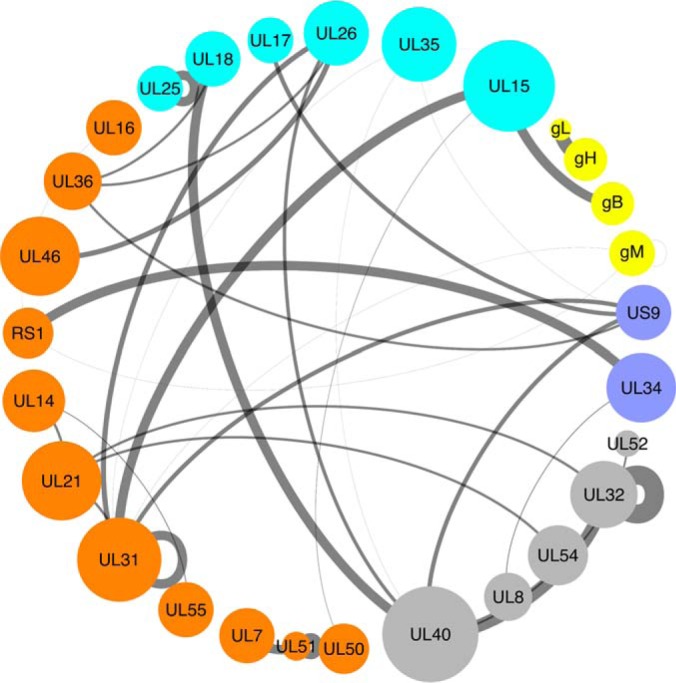
**Subnetwork highlighting homologous PPIs with a MIscore > 0. 4 (supplemental Table S2).** The node size indicates the number of interacting partners for each protein (*degree*). Edge width is scaled according to the confidence score associated with the PPI. The nodes are color-coded according to the protein location in the virion particles - gray: protein has not been detected in virions by MS in Loret *et al.* (34); cyan - capsid and capsid-associated protein; orange - tegument protein; yellow - envelope glycoprotein; dark blue - envelope protein (not glycoprotein).

The resulting data set, referred hereafter as *high-confidence homologous interactions subset*, comprises a total of 35 interactions (30 hetero-interactions and 5 self-interactions) among 28 different proteins (Fig. 5, supplemental Table S1). 28 hetero-interactions out of the 30 are connected in a *single connected component*, *i.e.* a group of nodes in which a path can be traced for any random pair of nodes (68). These interactions involve 23 proteins that have been annotated as being involved mainly in transcription, capsid assembly, and nuclear capsid egress events (supplemental Table S2). The two binary interactions outside the main graph involve the obligate hetero-dimer gH/gL, an essential component of the herpesvirus entry machinery (69), and the tegument proteins pUL7 and pUL51. The minor capsid protein pUL25 is also present in the data set with only one self-interaction. It is worth emphasizing that these disconnected components are nevertheless connected to the rest of the network when the whole interactome is considered (*i.e.* without filtering interactions). Our HVint interactome highlighted envelope protein pUS9 to be a hub for several potential interactions. Its predicted first neighbors, including VP26 (pUL35), the major small capsid protein (SCP), pUL17, another minor-capsid protein that interacts with pUL25, and the inner tegument protein pUL36, that are known to function in capsid structure, maturation, and tegument association (70–74). As the function of these proteins has not been studied in the context of pUS9-mediated virion maturation and transport, they represent promising targets for future investigation.

##### Validation of New Interactions

To assess the informative value of our novel PPI network (Fig. 5), we first searched the published literature for experimental evidence that had not been used as an input to our HVint (*i.e.* has yet to be incorporated within one of our source databases). We found recent experimental evidence supporting a complex formation of tegument proteins pUL7 and pUL51; coimmunopurification followed by MS detected this interaction, which was then confirmed and functionally characterized (75).

**Fig. 6.**
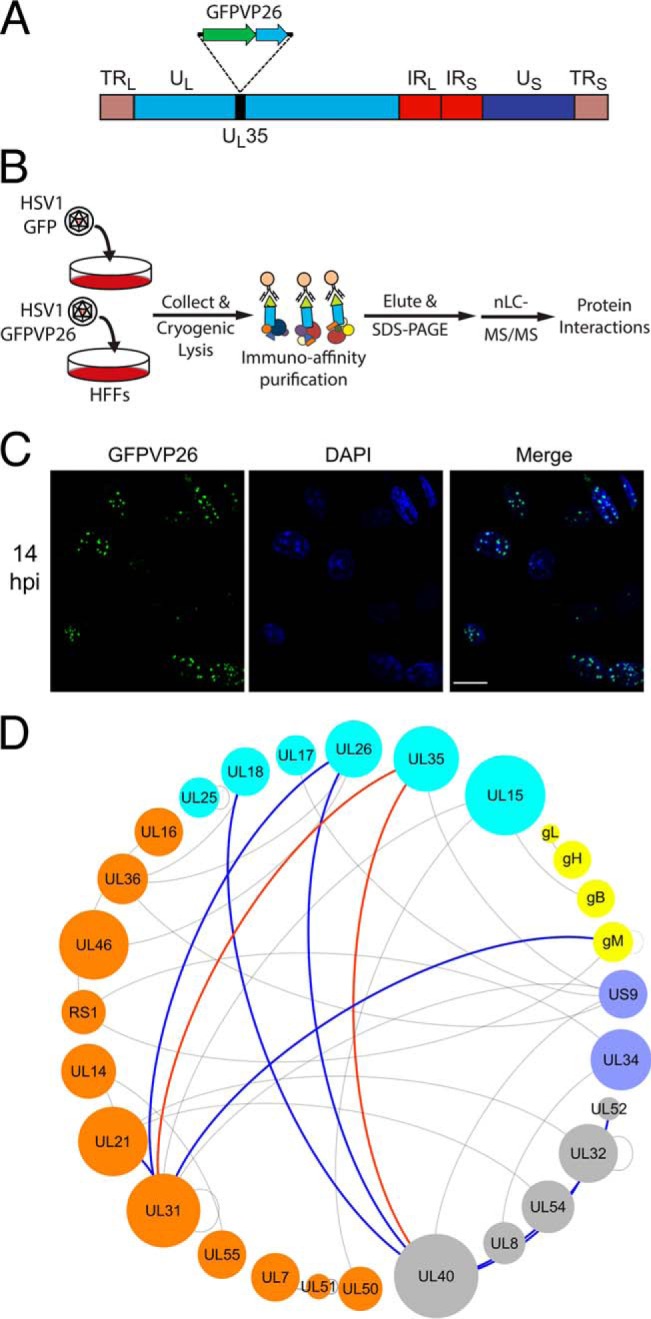
**Validation of homologous PPIs by IP-MS.** *A*, Schematic of the HSV-1 (strain 17^+^) genome indicating the locus (UL35 gene) that encodes for GFP-tagged VP26. *B*, Experimental design for IP-MS validation of predicted VP26 protein interactions. Primary human fibroblasts were infected with HSV1 GFP or HSV1 GFPVP26, then collected and cryogenically lysed. GFPVP26 and control GFP were immuno-affinity purified from cell lysates using α-GFP antibodies conjugated to magnetic beads. Coisolated proteins were eluted from the beads and resolved by SDS-PAGE, followed by nLC-MS/MS analysis. *C*, Fluorescence microscopy of human foreskin fibroblasts infected with HSV1-GFPVP26. The cellular localization of GFP-tagged VP26 was visualized by direct fluorescence (green) at 14 h post-infection (hpi). Nuclei were stained by DAPI (blue). Scale bar = 20 μm. *D*, The HVint subnetwork of homologous predicted PPIs (Fig. 5) was annotated to indicate PPIs that were coisolated with VP26 (pUL35) by IP-MS. Both direct (red edges) and indirect, secondary (blue edges) PPIs are highlighted.

Second, we conducted affinity purification MS-based proteomics experiments. From the homology-based network, we selected VP26 (pUL35) for affinity purification from a productive HSV-1 infection as a respective replication-competent GFP-tagged virus was available (76). Primary human fibroblast cells were infected with HSV1-GFP or HSV1-GFPVP26, which are HSV-1 (17^+^) strains expressing EGFP alone (control) or EGFP-tagged VP26, respectively (c.f. Experimental Procedures and (76)). Wide field fluorescence microscopy was used to determine the subcellular localization of GFPVP26 following infection (Fig. 6). We focused on 14 h post infection (p.i.), a relatively late time point in infection when under our experimental conditions capsid and virus assembly are in progress (Fig. 6*C*). Thus, this represents the stage of infection when VP26 is predominantly nuclear, and expected to interact with other capsid components. Additionally, at this stage, capsids containing VP26 are also starting to undergo nuclear export, and thereby expected to associate with proteins involved in capsid egress. Both of these subsets of interactions, capsid-associated and nuclear egress proteins, were part of our predicted VP26 interactions (Fig. 5). The GFP tag was used for affinity isolation of GFPVP26 using specific, high-affinity anti-GFP antibodies under relatively stringent conditions (60).

The immuno-isolated proteins from cells infected with HSV1-GFP or HSV1-GFPVP26 were digested with trypsin and analyzed by liquid chromatography-tandem MS. Viral proteins detected with at least fivefold greater spectral counts in HSV1-GFPVP26 compared with the HSV1-GFP control immuno-isolations were considered high confidence candidate interactions (supplemental Table S3). Although VP26 was isolated here using another HSV-1 strain and different lysis conditions when compared with a previous study ((60); c.f. Experimental Procedures), 90% of the enriched interaction candidates were the same, pointing to reproducible findings. Importantly, our experiment confirmed two of the three predicted direct PPIs of VP26, specifically with pUL31 and pUL40 (Fig. 6*D*, red edges). The third predicted association with pUS9 was the only predicted direct association not detected under these lysis conditions so far. However, we cannot exclude the possibility that pUS9 is a bona fide interaction that was not maintained in the applied lysis buffer. Given the relatively small size of US9 (∼10 kDa and 90 amino acids), its detectability by mass spectrometry would be more challenging than either pUL31 or pUL40. In fact, an *in silico* tryptic digestion of HSV1 US9 predicts only 1 - 3 tryptic peptides to be detected under our instrument configuration and methodology.

Because immuno-affinity purification of GFPVP26 is expected to also identify indirect interacting partners, the proteins coisolated with GFPVP26 were compared with the predicted, second order VP26 interactions (via pUL40 and pUL31, see Fig. 5). Indeed, the IP-MS experimental data set identified the proteins pUL32, pUL18, pUL26, pUL52, pUL21, and pUL10 (gM). Binary interactions between pUL40 and each of pUL32, pUL18, pUL26, and pUL52, as well as between pUL31 and each of pUL26, pUL21 and gM are predicted by our high-confidence homologous HVint subnetwork (MIscore > 0.4). Additionally, the interaction between pUL31, a nuclear egress protein, and pUL32, a protein suggested to contribute in efficient localization of newly synthesized capsids to nuclear replication compartments and DNA packaging, is present in our homology-transferred data set but with scores below the 0.4 cut-off. Overall, the experimental immuno-affinity purification experiments provide compelling support for the homologous HVint network.

##### Interactive Web-based HVint Interface

All the data in HVint, including pairwise interactions, lines of evidence, and scores, are available for download in a standard format for molecular interactions data, namely PSI-MITAB 2.5. Additionally, we provide a user-friendly and intuitive interface to browse the data in a more straightforward and intuitive manner (http://topf-group.ismb.lon.ac.uk/hvint). The HVint interface displays an interactive graphical representation of the network and allows easy access to annotation data for individual nodes and edges. These can be selected by clicking on them or, in the case of nodes, specifying their UniProt ID in the provided search tool. The subnetwork created by the first neighbors of the selected element is then presented, together with its associated data (including a list of interactions, confidence scores and supporting evidence) in tabular form. Other functionality of the interface includes the ability to filter interactions based on user-defined confidence thresholds.

## DISCUSSION

HVint was originally populated with data from five major external public resources. Although some overlap exists in the data coming from these databases, each contributes multiple unique interactions. A recurrent issue in interactomics studies is the lack of network coverage, which severely limits the informative power of the resulting networks (77, 78). Therefore, in retrieving data from the five resources, we distinguished between two data sets. One data set contained native interactions reported as directly detected for HSV-1, and the second one was composed of homologous interactions detected in any of the other human herpesvirus species, covering all three herpesvirus subfamilies. The latter data set was then used to predict PPIs in HSV-1 based on orthology relationships between the interacting partners in the homologous species and HSV-1 proteins. Integrating these data increased the network coverage by more than twofold. More importantly, it provides a set of putative novel interactions to be validated in HSV-1. To provide a measure of the quality and reliability of the data in the database, we implemented the MIscore (53) scoring function in our protocol. MIscore integrates data from multiple experiments that report a given interaction to calculate an overall confidence score, which is normalized to the interval [0, 1]. It calculates weighted scores based on key variables: experimental detection method(s) (*e.g.* biophysical, imaging), type of interaction (*e.g.* physical association, colocalization), and number of different scientific publications reporting it. In our implementation we gave lower weighting to orthology-transferred interactions by penalizing their original score in a sequence-identity dependent manner. An advantage of implementing this scoring scheme was that it yielded a calibrated series of scores (ranging from 0 to 1), which allowed for fine-tuned selection of sets of interactions based on their confidence levels. We used this approach to select a subset of high-confidence interactions for further experimental testing.

Overall, our interactome, including 419 interactions, achieved a notably larger coverage than any individual source database alone. This set is split between 195 native and 255 homologous interactions, with a small subset of them overlapping between the two (31 interactions), and it covers all 77 proteins in the HSV-1 reference proteome (as reported in UniProt (48)). The data integration framework used in this study (Fig. 1) is automated and relatively simple, which will allow future updates to be implemented easily as new interaction data becomes available.

The prevalence of Y2H experiments in protein-protein interaction studies (79) is reflected in the large fraction of interactions obtained by this method in HVint (∼90%, 380 interactions). This is because of the ability of Y2H experiments to determine physical pairwise interactions between potentially interacting partners, and from the feasibility of conducting both small- and large-scale screenings. Since the publication of the first large-scale protein interactions map for *S. cerevisiae* in 2000 (80), multiple studies have followed and several caveats have been raised regarding the interpretation and reliability of Y2H data, especially when obtained from high-throughput screenings (81). The main concern is the larger false positive and false negative rates than in previous traditional small-scale approaches. This has demanded a continued optimization of the technique (as shown in recent reports (79, 81–83)), and the development of a range of alternative hybrid strategies to complement Y2H data sets (79). These approaches have minimized the error rate in more recent data, suggesting that in the future the reliability of Y2H data could be assessed on a per data set basis in the future. HVint includes data derived from Y2H experiments across a broad period of time. Consequently, we could not yet, at present, systematically assess the quality of each data set (as this is not reflected in the source databases). The use of MIscore as a scoring method is advantageous in this respect; it assigns comparatively low scores to interactions detected by Y2H experiments, thereby representing a conservative approach in assessing the confidence of these interactions.

To test the predictive power of our computationally derived interaction data set, we subjected a subset of PPIs with high-confidence scores to experimental validation. To this end, a number of homologous-only interactions that scored above 0.4 were selected. We focused our own validation analysis on VP26 (encoded by UL35), because it is a protein whose functions are still unclear, and because several HSV-1 strains in the F, 17^+^ and KOS background have been generated, in which VP26 has been successfully tagged with fluorescent proteins without or with minor impairment of HSV-1 propagation (58, 60, 84–87). Hexamers of VP26 are located on top of the capsid hexons, which are hexamers of VP5, but not on the pentons, which are pentamers of VP5. VP26 is therefore perfectly placed on the capsid surface for interactions with other viral and cellular components (74, 76, 88, 89). Mutants of pseudorabies virus (PRV), a porcine α-herpesvirus, or of HSV-1, that lack VP26, are less neuroinvasive and neurovirulent and grow to lower titers than their respective parental strains (85, 90–92). VP26 can interact with the dynein light chains of the Tctex family in Y2H and coIP experiments. However, it is not required for efficient dynein-mediated transport toward the nucleus during cell entry, and dynein does not bind to un-tegumented capsids that expose VP26 over their entire surface (74, 76, 84, 93). Homologous small capsid proteins of other herpesviruses have been shown to contribute to capsid stability (94, 95).

**Fig. 7.**
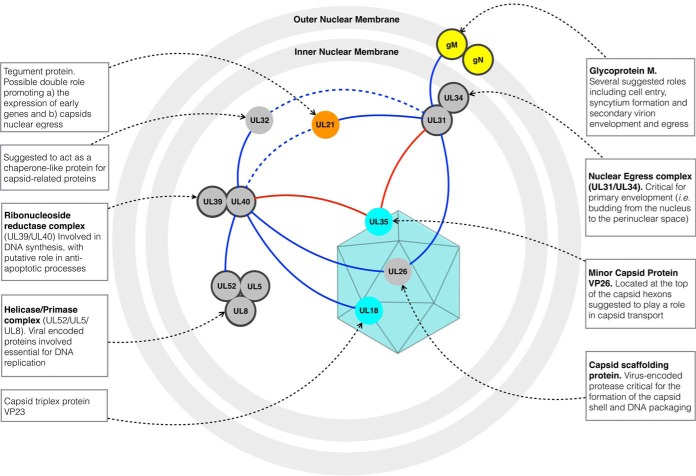
**Novel interactions predicted by HVint and supported by IP-MS experiments (Fig. 6) in the context of the viral life cycle.** Proteins are color-coded according to the protein location in the virion particles - gray: protein has not been detected in virions by MS in Loret *et al.* 2008 (34); cyan - capsid and capsid-associated protein; orange - tegument protein; yellow - envelope glycoprotein. Solid lines indicate predicted interactions in HSV-1 with MIscore > 0.4; dashed lines indicate predicted interactions with MIscore ≤ 0.4. Both direct (red edges) and indirect, secondary (blue edges) PPIs are highlighted.

Our HVint homologous subnetwork predicts that VP26 is associated with pUL31 and pUL40 (Fig. 7). This is supported by our experimental immuno-affinity isolation studies. VP26 is present in the mature virion particle, whereas pUL31 and pUL40 are not part of it (34, 88, 89, 96–99). Hence these interactions may be particularly relevant in understanding the role of VP26 in the viral life cycle. Notably, VP26 was also coisolated with pUL34, one of the known binding partners of pUL31 (supplemental Table S3). Together, pUL31 and pUL34 form the so-called nuclear egress complex, which is anchored to the inner nuclear membrane by the C-terminal domain of pUL34 and faces the nuclear lumen (99, 100). Given the conservation of the interaction between pUL31 and pUL34 and their respective orthologs across the entire *Herpesviridae* family (101), many studies have explored their roles in the early steps of the capsid assembly and the nuclear egress pathway. Besides being recruited directly to pUL34 at the inner nuclear envelope, pUL31 has also been reported to interact via its N-terminal domain to newly synthesized nuclear capsids (102). The pUL31-bound capsids are then thought to be translocated to the inner nuclear membrane, where they associate via UL31 with pUL34 to mediate capsid egress from the nucleoplasm to the cytosol (102). Overall, the localization of VP26 to the capsid surface except the vertices, its documented roles in capsid trafficking, and our computational and experimental findings of VP26 association with nuclear egress and CVSC complexes, suggests an as yet unknown role for VP26 in enforcing capsid stability and coordinating intra-nuclear capsid trafficking events (Fig. 7). Further, the recent *in situ* analyses of the NEC architecture revealed a curved hexagonal lattice for PRV (103, 104). As pUL31 and pUL34 are highly conserved in the *Herpesviridae* this architecture is highly likely to be evident in HSV-1 as well. Therefore, an attractive hypothesis is that VP26 hexamers and pUL31/34 hexamers interact directly with each other. Future, *in situ* analysis will need to show whether this is in fact the case.

Another pair of viral proteins, pUL39 and pUL40, which constitute the small and the large subunits (RR1 and RR2, respectively) of the heterotetrameric HSV-1 ribonucleotide reductase complex (105–108), were also coisolated with VP26 by IP-MS analysis. Intriguingly, the latter was also predicted to interact with VP26 in our homologous subnetwork. In addition to this enzymatic function, several studies have highlighted a role for pUL39 as a virulence factor (106, 108–110), for instance by interfering with apoptotic cascades (106, 109–111). This again suggests a potential role for VP26 in the early events of virus replication. Our experimental validation supports the interaction between VP26 via pUL40 to pUL39 and further to each of pUL32, pUL18 (VP23) and one or more products of the HSV-1 UL26 gene, which encodes for an autocatalytic protease that is processed into the scaffold proteins VP24 and VP21 (112, 113). Notably, all these interactions have been predicted in our homologous subnetwork. Further, a recent study has provided new insights into a possible role of pUL32 as a chaperone-like protein capable of modulating capsid maturation and tegument acquisition by interacting with the capsid proteins pUL6 (the portal protein), pUL25, and pUL38 (VP19c) as well as the inner tegument protein pUL36 (114).

Among the membrane proteins, our HVint interactome provides a particularly high number of novel interactions for pUS9. pUS9 is a small type II membrane protein of about 10 kDa that localizes to the trans-Golgi network and axonal vesicles of neuronal cells (115–117). Our HSV-1 interactome predicts interactions between pUS9 and the capsid proteins VP26 and pUL17, as well as the inner-tegument protein pUL36. pUL17 and pUL25 form the Capsid Vertex-specific component (CVSC) located on the pentons edges and pUL17 and pUL25 are both required for stable DNA packaging into capsids prior to nuclear egress (70, 118, 119). pUL36 is an inner protein known to be involved in early tegumentation events together with its binding partner pUL37 (72, 84). Like the CVSC, the complex pUL36-pUL37 is also located around penton vertices. Interestingly, PPIs and coordinated events between pUL36-pUL37 and CVSC have been previously shown (72). Together with the heterodimer gE/gI, pUS9 mediates anterograde axonal transport of viral tegument, glycoproteins, and virions of α-herpesviruses in neurons, but these proteins are not essential for replication in epithelial or fibroblasts cells (40–42). Although studies on the gE/gI and pUS9-mediated transport of PRV structures show that PRV is transported along axons as fully assembled virions in transport vesicles, the data on HSV-1 regarding the subviral and viral particles being targeted to axons is still controversial (116, 117, 120). Because the HSV-1 or PRV proteins that might contribute to these differences are not known, our novel predicted interactions from HVint generate new hypotheses on potentially virus-specific regulators of US9-dependent anterograde transport.

The nodes outside the main cluster of the 35 new interactions involve those forming two heterodimers conserved across the entire *Herpesviridae* family, namely gH/gL and pUL51-pUL7, and one self-interaction for pUL25. The complex gH/gL was structurally solved in HSV-2 by x-ray crystallography in 2010 (121). Given the high confidence of the experimental data and the degree of sequence similarity between HSV-1 and HSV-2 gH/gL sequences, the existence of this complex in HSV-1 is undisputed (121–123). Although its specific role is yet to be fully defined, it is likely involved in regulating the activity of the membrane fusion glycoprotein gB (121).

Finally, complex formation between pUL51 and pUL7, as predicted in our HVint database, was confirmed recently by affinity purification and tandem MS experiments (75). At the time of writing, these data were not integrated into the source PPI databases used in this work. pUL51 and pUL7 are both conserved across the whole *Herpesviridae* family. pUL51 is an N terminus membrane-anchored tegument protein (124–126) with no apparent enzymatic function, suggested to play a role in viral egress, viral envelopment and epithelial cell-to-cell spread (127, 128). Its interaction with pUL7 seems to be required for efficient recruitment of the latter to cytoplasmic membranes leading to its incorporation into the virion. Both proteins partially colocalize in cytoplasmic membranes together, as observed in the case of pUS9, with glycoprotein gE (128). This implies that the complex is non-obligate and each protein could carry out independent functional activities. pUL7 was also found inside the nuclear and cytoplasmic compartments in the absence of pUL51, and interactions between pUL7 and capsid proteins have been reported. As in the case of pUS9, the complex between pUL51 and pUL7 supports the idea that viral membrane proteins are principal agents in tegumentation and secondary envelopment processes. By establishing interactions with capsid and capsid-associated proteins they guide progeny virion particles to different cytoplasmic compartments (*e.g.* trans Golgi network or endosomes) until they reach the plasma membrane (42, 129–134).

## CONCLUSIONS

Here we introduce the HVint database, an integrative resource for HSV-1 protein interaction data. The database contains interactions experimentally detected in HSV-1 species and computationally predicted using evolutionary information. Interactions in the database have been scored, thus confidently providing novel predicted interactions. Consequently, HVint can be used as a tool to prioritize candidate interactions for future experimental testing. The protocol used to create and populate the database was kept simple, hence facilitating database updates. In the future, our aim is to further improve our methods for assessing the reliability of protein interaction data and to include more advanced features in the HVint graphical interface to accommodate users' needs. We also plan to extend the current database to other human herpesvirus species and to virus-host interactions.

## Supplementary Material

Supplemental Data

## Footnotes

^1^ The abbreviations used are:

Co-IP: Co-Immunoprecipitation
PPI: protein-protein interaction
Y2H: Yeast Two-Hybrid
NMR: nuclear magnetic resonance
EMDB: Electron Microscopy Data Bank
HSV-1: HHV-1, herpes simplex virus type 1, human herpesvirus 1
GFP: Enhanced Green Fluorescent Protein
DIP: Database of Interacting Proteins
HSV-2: HHV-2, herpes simplex virus type 2, human herpesvirus 2
VZV: HHV-3, varicella-zoster virus, human herpesvirus 3
EBV: HHV-4, Epstein-Barr virus, human herpesvirus 4
HCMV: HHV-5, human cytomegalovirus, human herpesvirus 5
HHV-6: human herpesvirus 6
HHV-7: human herpesvirus 7
KSHV: HHV-8, Kaposi's sarcoma-associated herpesvirus, human herpesvirus 8
NCBI: National Center for Biotechnology Information
EMBL: European Bioinformatics Institute
DDBJ: DNA Data Bank of Japan
PDB: Protein Data Bank
HMM: Hidden Markov model
PSI: Proteomics Standards Initiative
MI: molecular interactions
CVSC: capsid vertex-specific component
PFU: plaque forming unit.
