# Supplementary material for: HVint: A Strategy for Identifying Novel Protein-Protein Interactions in Herpes Simplex Virus Type 1
Source: Mol Cell Proteomics. 2016 Jul 6;15(9):2939–53. doi: 10.1074/mcp.M116.058552 (PMC5013309; doi:10.1074/mcp.M116.058552)
Supplement: Supplemental Data [file supp_15_9_2939__index.html]

HVint: A strategy for identifying novel protein-protein interactions in herpes simplex virus type 1 — HVint: A Strategy for Identifying Novel Protein-Protein Interactions in Herpes Simplex Virus Type 1 — New Protein-Protein Interactions in HSV-1 — Supplemental Data 

# HVint: A Strategy for Identifying Novel Protein-Protein Interactions in Herpes Simplex Virus Type 1

## Supplemental Data

- Tables\_S1,2\_and\_Figures\_S1,2,4 (.pdf, 1.3 MB) - Tables\_S1,2\_and\_Figures\_S1,2,4
- Figure\_S3 (.html, 7 KB) - Figure\_S3
- Table\_S3 (.xlsx, 179 KB) - Table\_S3
