# Supplementary material for: HVint: A Strategy for Identifying Novel Protein-Protein Interactions in Herpes Simplex Virus Type 1
Source: Mol Cell Proteomics. 2016 Jul 6;15(9):2939–53. doi: 10.1074/mcp.M116.058552 (PMC5013309; doi:10.1074/mcp.M116.058552)

Supplementary table 1: List of homology transferred protein-protein interactions (PPI) with confidence score > 0.4.

Supplementary table 2: List of proteins involved in the subset of high confidence homology transferred protein-protein interactions (Supplementary table 1). Functional description based on UniProt entry annotation.

Supplementary table 3: Tables of viral proteins that were either unique or enriched in GFPVP26 (N = 3) versus GFP HSV1 control (N = 3) affinity isolations. Interactions predicted by HVint and supported by the GFPVP26 isolations are indicated in red text (pUL40 and pUL31). The tables report each protein's description, gene symbol, UniProt accession, molecular weight (MW, kDa), spectral count fold enrichment versus GFP ("Viral Int, FC vs GFP") or unweighted spectral counts and MS1-based label-free quantitative (for GFPVP26 IP) ("Viral Int, SC"). A column was also included to indicate if the viral protein was co-isolated with VP26 in Rowles et al. publication (62). Additional sheets with the above details list the co-isolated host proteins ("Host Int") and proteins that did not meet the IP specificity filters ("Filtered_Non-specific").

Supplementary figure 1: Venn diagram showing the distribution of PPIs based on the type of experimental evidence. Empty subsets indicate zero overlap.

Supplementary figure 2: PPI sub-network involving only proteins present in extracellular virion particles. The node size indicates the number of interacting partners for each node (*degree*). Edge width is scaled according to the confidence score associated with the PPI. Nodes are colour-coded according to the protein location in the virion particles – cyan – capsid and capsid-associated protein; orange – tegument protein; yellow – envelope glycoprotein; dark blue – envelope protein (not glycoproteins).

Supplementary figure 3: Interactive plot presenting the initial (cyan) and final (magenta) scores derived from MIscore (initial scores) for homologous PPIs with an amino acid sequence identity of less than 40%. The final scores were computed by applying a penalising function to the original scores in a sequence-identity dependent manner (see Methods).

Supplementary figure 4: Scatter plots showing the changes of several network properties after filtering interactions based on gradually increasing confidence cut-offs. **(A)** Average path length, node degree, connected components; **(B)** Clustering coefficient, centralisation, density and heterogeneity.

Supplementary table 1. (Location: Results – New Interactions)

**Source** (UniProt)	**Target** (UniProt)	**Source** (ORF)	**Target** (ORF)	**Interaction type**	**Confidence score**	**Present in virion**
P10216	P10216	UL32	UL32	Homo-interaction	0.712	No
P06477	P10185	gH	gL	Hetero-interaction	0.623	Yes
P10216	P10224	UL32	UL40	Hetero-interaction	0.609	No
P10235	P10191	UL51	UL7	Hetero-interaction	0.553	Yes
P10235	P10235	UL51	UL51	Homo-interaction	0.553	Yes
P10209	P10209	UL25	UL25	Homo-interaction	0.553	Yes
P10224	P10202	UL40	UL18	Hetero-interaction	0.553	No
P08392	P10218	ICP4	UL34	Hetero-interaction	0.553	No
P10215	P04295	UL31	UL15	Hetero-interaction	0.553	No
P10215	P10215	UL31	UL31	Homo-interaction	0.553	No
P10211	P04295	gB	UL15	Hetero-interaction	0.553	No
P10210	P10230	UL26	UL46	Hetero-interaction	0.484	No
P10215	P10210	UL31	UL26	Hetero-interaction	0.484	No
P06481	P10201	US9	UL17	Hetero-interaction	0.470	Yes
P10224	P06481	UL40	US9	Hetero-interaction	0.470	No
P10215	P06481	UL31	US9	Hetero-interaction	0.470	No
P10224	P10210	UL40	UL26	Hetero-interaction	0.456	No
P04291	P10205	UL14	UL21	Hetero-interaction	0.442	Yes
P10202	P10220	UL18	UL36	Hetero-interaction	0.442	Yes

P06481	P10220	US9	UL36	Hetero-interaction	0.442	Yes
P10210	P10220	UL26	UL36	Hetero-interaction	0.442	No
P10205	P10238	UL21	UL54	Hetero-interaction	0.442	No
P10205	P10215	UL21	UL31	Hetero-interaction	0.442	No
P10205	P10216	UL21	UL32	Hetero-interaction	0.442	No
P10236	P10224	UL52	UL40	Hetero-interaction	0.432	No
P04291	P10239	UL14	UL55	Hetero-interaction	0.415	Yes
P10192	P10218	UL8	UL34	Hetero-interaction	0.415	No
P10234	P04295	UL50	UL15	Hetero-interaction	0.409	No
P08392	P10200	ICP4	UL16	Hetero-interaction	0.401	Yes
P04288	P08392	gM	ICP4	Hetero-interaction	0.401	Yes
P04288	P04288	gM	gM	Homo-interaction	0.401	Yes
P06481	P10219	US9	UL35	Hetero-interaction	0.401	Yes
P10224	P10219	UL40	UL35	Hetero-interaction	0.401	No
P10215	P10219	UL31	UL35	Hetero-interaction	0.401	No
P10215	P04288	UL31	gM	Hetero-interaction	0.401	No

Supplementary table 2. (Location: Results – New Interactions)

**Node** (UniProt)	**Common name / function**	**ORF**
P04295	Tripartite terminase subunit pUL15	UL15
P10200	Capsid-binding protein pUL16	UL16
P10224	Ribonucleoside-diphosphate reductase small subunit pUL40	UL40
P10220	Deneddylase and large tegument protein (LTP) pUL36	UL36
P10230	VP11/12 (pUL46)	UL46
P10219	Capsid protein VP26 (pUL35)	UL35
P10192	DNA helicase/primase complex-associated protein pUL8	UL8
P10210	Protease precursor pUL26 (cleaved to VP24 and VP22a)	UL26
P08392	Major viral transcription factor ICP4	RS1
P10238	Transcriptional regulator ICP27	UL54
P04291	pUL14	UL14
P04288	Glycoprotein M (gM)	UL10
P10201	Virion-packaging protein pUL17 (member of CVSC)	UL17
P10205	pUL21	UL21
P10218	pUL34	UL34

P10215	pUL31	UL31
P10202	Triplex capsid protein VP23 (pUL18)	UL18
P10239	pUL55	UL55
P10191	pUL7	UL7
P10216	Packaging protein pUL32	UL32
P10236	DNA primase pUL52	UL52
P10235	pUL51	UL51
P10211	Glycoprotein B (gB)	UL27
P10209	Virion-packaging protein pUL25 (CVSC)	UL25
P06481	US9	US9
P10234	dUTPase pUL50	UL50
P06477	Glycoprotein H (gH)	UL22
P10185	Glycoprotein L (gL)	UL1

Supplementary table 3. (Location: Results – Validation of New Interactions; Discussion)

Separate Microsoft Excel file.

Supplementary figure 1. (Location: Experimental Procedures – Homology Transfer of Interactions)

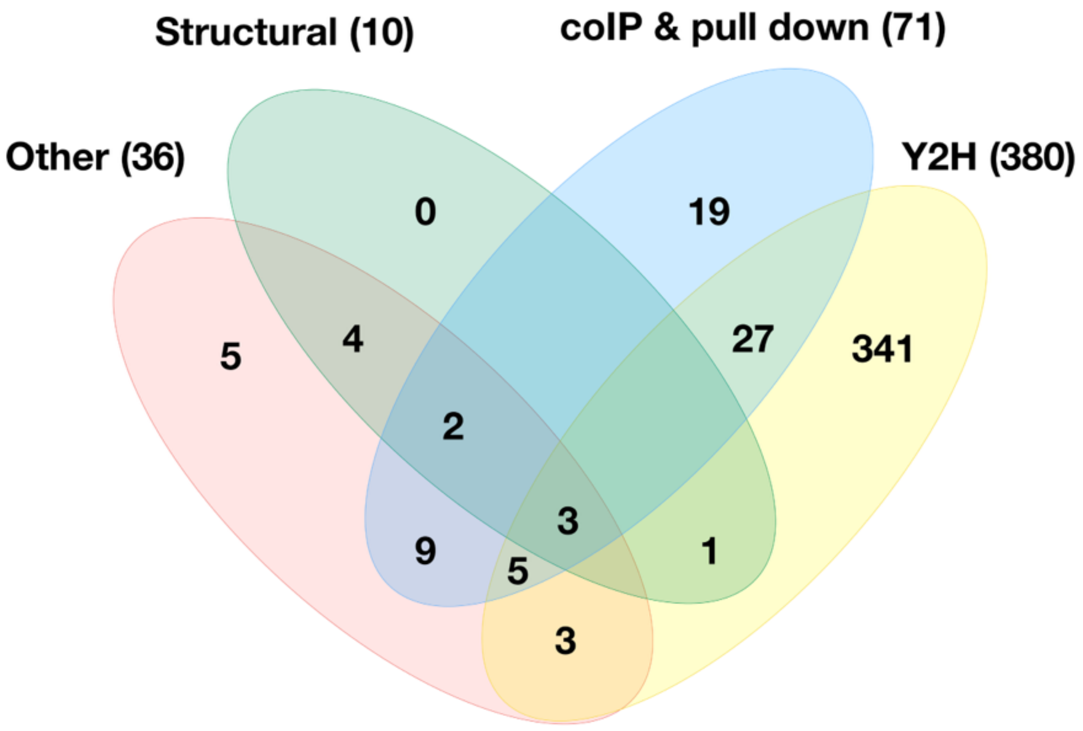

Supplementary figure 2. (Location: Experimental Procedures – Homology Transfer of Interactions)

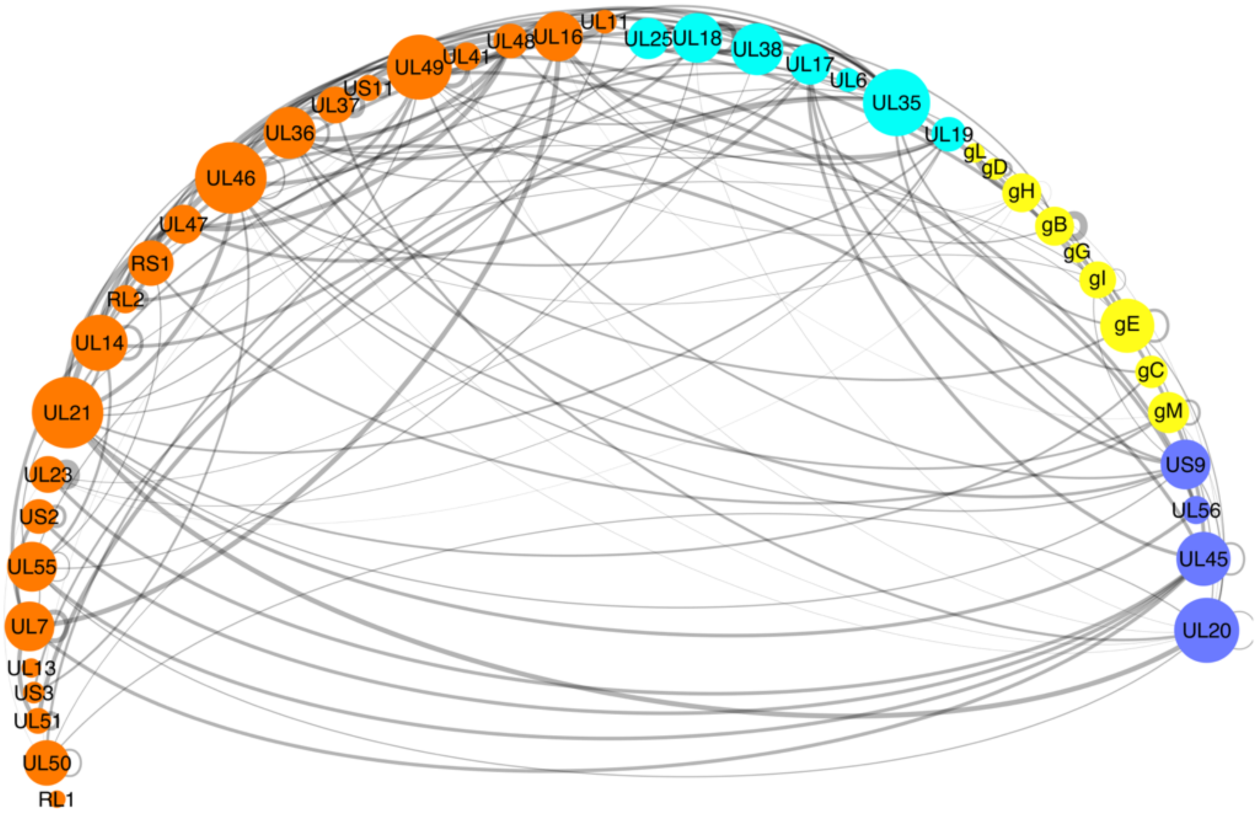

Supplementary figure 3. (Location: Experimental Procedures – Scoring of Interaction Data)

Separate HTML file.

Supplementary figure 4. (Location: Results – New Interactions)

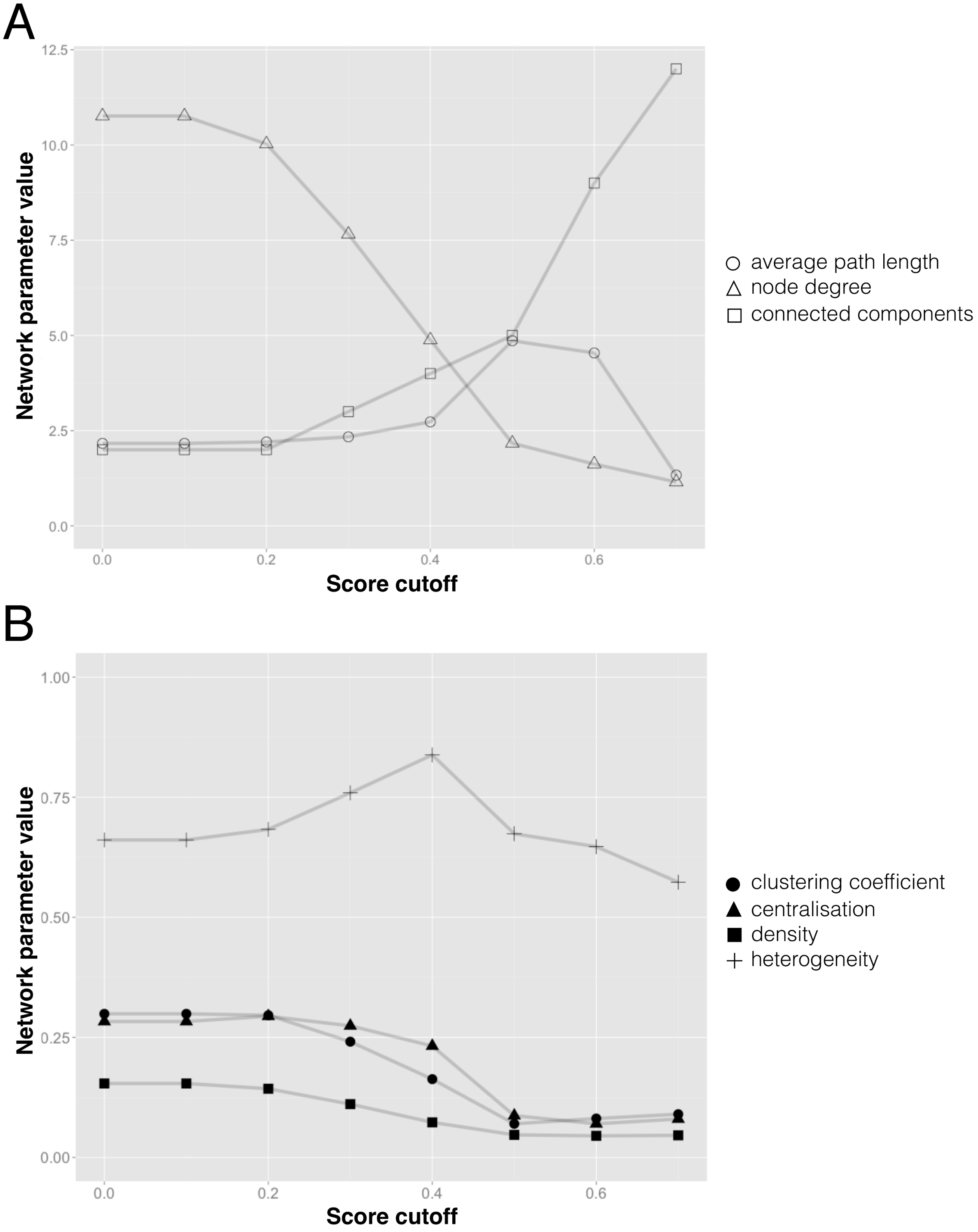

Supplement: Supplemental Data [file 10.1074_M116.058552_mcp.M116.058552-1.pdf]
