# Supplementary material for: HVint: A Strategy for Identifying Novel Protein-Protein Interactions in Herpes Simplex Virus Type 1
Source: Mol Cell Proteomics. 2016 Jul 6;15(9):2939–53. doi: 10.1074/mcp.M116.058552 (PMC5013309; doi:10.1074/mcp.M116.058552)
Supplement: Supplemental Data [file 10.1074_M116.058552_mcp.M116.058552-2.html]

mcp.M116.058552-2 

# HVint: A Strategy for Identifying Novel Protein-Protein Interactions in Herpes Simplex Virus Type 1
